# Editorial: Achieving Well-Being—Bridging Psychological Distance in Our Environment

**DOI:** 10.3389/fpsyg.2022.813341

**Published:** 2022-03-17

**Authors:** Zepeng Tong, Yan Sun

**Affiliations:** ^1^Key Laboratory of Behavior Science, Institute of Psychology, Chinese Academy of Science, Beijing, China; ^2^Department of Psychology, University of Chinese Academy of Science, Beijing, China

**Keywords:** psychological distance, environment, well-being, construal level, green consumption

## Bridging Psychological Distance in Our Environment

Current research suggests that the alienation between people creates psychological distance which leads to the rejection of individual responsibilities of protecting the environment (Kim and Wolinsky-Nahmias, 2014; Wang et al., 2021). Moreover, the rapidly growing environmental issues have harmed people's safety, decreasing public well-being. For those reasons, it is urgent to reduce the psychological distance between citizens and severe environmental problems (such as climate change) to improve the pro-environmental behavior intentions of people.

The concept of psychological distance can be described as the relationship between an individual and a specific object or event (Trope and Liberman, 2003). When the object is perceived as psychologically close, it is represented as being more concrete and authentic, while when the object is perceived as psychologically distant, the representation is more abstract (Liberman and Trope, 2008). Past research revealed that psychological distance could be involved in environmental issues. For instance, people will have a more abstract representation of climate change when they perceive it as more distant (McDonald et al., 2015). By comparison, individuals are more likely to behave in favor of the environment when they perceive the problems of environmental deterioration as having direct consequences for themselves (Lorenzoni and Pidgeon, 2006). Following the previous conclusions, narrowing the psychological distance between the general public and environmental problems is a management strategy for climate change or other environmental issues worthy of consideration.

The current Research Topic aims to advance understanding of the psychological distance between the public and environmental issues at theoretical and practical levels. The validated countermeasures from research of the present topic will provide correct guidance for citizens to develop responsible environmental behaviors and to reach a higher level of life satisfaction and well-being. For policymakers or non-governmental organizations, these findings will help them formulate more appropriate management strategies or more effective public campaign projects.

## Influences of Psychological Distance on Environmental Attitude and Behavior

From the perspective of psychological distance, the public tends to think that the damage brought by environmental problems may soon occur to people in other regions or countries (Kollmuss and Agyeman, 2010). Applying these insights, Xu et al. explored how the social member's perceived psychological distance affects their willingness to spread pro-environment behaviors; when social members perceived they had a close psychological distance to environment change, their willingness to spread pro-environment behaviors also increased. Maiella et al. conducted a systematic review on psychological distance and climate change. Their work showed that when individuals perceived climate change as more proximal and concrete within the construct of psychological distance, they had a higher propensity to perform pro-environmental behaviors. Sun et al. reported how people coped with negative emotions in response to the epidemic from the perspective of psychological distance. The longitudinal result showed that independent information effectively decreased fear and anxiety, while interdependent information effectively mitigated sadness. Sheng, Dai et al. discussed the influence of air quality on pro-environmental behavior. They revealed that air pollution within the local spatial distance could make individuals more willing to conduct pro-environmental behavior. Similarly, Liu et al. explored how individuals' psychological distance toward air pollution influences their purchase intentions for new energy vehicles. Their findings suggest that closer psychological distance toward air pollution is accompanied with a stronger intention to purchase new energy vehicles.

## Effects of Contextual Determinants on Psychological Distance

To consider the impact of the cognitive determinants on psychological distance, Tong, Li et al. proved that the anthropomorphic features would affect individuals' green purchase intentions. Moreover, the green trust played a mediating role between the anthropomorphic features and green purchase intention, which means anthropomorphic features could reduce the psychological distance between individuals and the green brand. Lee and Chen evaluated the effects of wearing masks on interpersonal space perception. A smaller interpersonal space was identified when individuals faced peers wearing masks than in the mask-free condition. Wei et al. indicated that individuals' recycling efforts could affect recycling behaviors. For costly recycling behaviors, those requiring physical or mental efforts will receive more attention due to the closer psychological distance. Chen et al. analyzed the psychological distance between people and climate change in the context of digital technology. The findings suggest that online activities bring climate deterioration closer to individuals through the visualization by digital technology, which can encourage individuals to participate in global climate cooperation.

## Practical Progress in Bridging Psychological Distance to Achieving Well-Being

To achieve the well-being of the general public, the present research aims to explore the practical application of psychological distance into the real world. Mi et al. studied the effect of personal-organization fit on employees' green behavior. They revealed the impact of personal-organization fit on employees' green behavior is enhanced in the case of close emotional expectation distance. In addition, Sheng, Xia et al. discussed green advertising from the perspective of spatial distance, suggesting that search products (or experience products) could enable consumers to generate a more positive attitude when the environmental aspect of the product was presented with close-up shots (or full-length shots). Ge et al. found that social norm conflict in green consumption created alienation among people by making individuals feel powerless and meaningless, in turn reducing their inclination toward green consumption. Tong, Liu et al. reported that the psychological distance moderating information framework and green product consumption willingness. For enterprises, their findings suggested if the target market was located in a region with more environmental problems (which means a closer psychological distance), the benefits of the products should be emphasized. Feng et al. tested the mediation of psychological distance between green housing buyer comments and purchase intentions. More importantly, in the purchase of green housing in the long run, psychological distance plays a more significant role than the prices.

## Future Directions

Taking together, it is clear from the articles on the current topic that the study of the psychological distance between the public and environmental issues is blossoming. The contributions of the present editorial cover a wide range of exciting new questions that span the theory, phenomenon, and governing strategy, which could speed up advances in the field of environmental psychology. The special issue explored the effects of various dimensions of psychological distance, but the distance of a stimulus on one dimension may influence its perceived distance on other dimensions (Liberman and Trope, 2008). Furthermore, pro-environment behaviors can be considered a multi-attribute decision task (Gong et al., 2020), which means the association of different dimensions of psychological distance will impact individuals' behavior. Thus, future research should contemplate questions about relationships among the various dimensions of psychological distance. Beyond this, from the perspective of practical management, succeeding research should conduct experiments in real scenarios to facilitate the ecological validity of results and interpretation of findings.

## Author Contributions

Both authors listed have made a substantial, direct, and intellectual contribution to the work and approved it for publication.

## Funding

This article was supported by grants from the Chinese Academy of Science Engineering Laboratory for Psychological Service (KFJ-PTXM-29) and the Institute of Psychology, Chinese Academy of Science (E1CX423003).

## Conflict of Interest

The authors declare that the research was conducted in the absence of any commercial or financial relationships that could be construed as a potential conflict of interest.

## Publisher's Note

All claims expressed in this article are solely those of the authors and do not necessarily represent those of their affiliated organizations, or those of the publisher, the editors and the reviewers. Any product that may be evaluated in this article, or claim that may be made by its manufacturer, is not guaranteed or endorsed by the publisher.
